# In “High-Risk” Infants with Sufficient Vitamin D Status at Birth, Infant Vitamin D Supplementation Had No Effect on Allergy Outcomes: A Randomized Controlled Trial

**DOI:** 10.3390/nu12061747

**Published:** 2020-06-11

**Authors:** Kristina Rueter, Anderson P. Jones, Aris Siafarikas, Ee-Mun Lim, Susan L. Prescott, Debra J. Palmer

**Affiliations:** 1 School of Medicine, The University of Western Australia, 35 Stirling Highway, Crawley, WA 6009, Australia; Kristina.Rueter@health.wa.gov.au (K.R.); Aris.Siafarikas@health.wa.gov.au (A.S.); susan.prescott@telethonkids.org.au (S.L.P.); 2 Perth Children’s Hospital, 15 Hospital Avenue, Nedlands, WA 6009, Australia; 3 InVIVO Planetary Health, Group of the Worldwide Universities Network (WUN), 6010 Park Ave, West New York, NJ 07093, USA; 4 Telethon Kids Institute, University of Western Australia, 15 Hospital Avenue, Nedlands, WA 6009, Australia; anderson.p.jones@gmail.com; 5 Department of Endocrinology and Diabetes, Sir Charles Gairdner Hospital, Nedlands, WA 6009, Australia; EEmun.Lim@health.wa.gov.au; 6 Department of Clinical Biochemistry, PathWest Laboratory Medicine, Queen Elizabeth II Medical Centre, Nedlands, WA 6009, Australia; 7 The ORIGINS Project, Telethon Kids Institute and Division of Paediatrics, University of Western Australia, 15 Hospital Avenue, Nedlands, WA 6009, Australia

**Keywords:** allergen sensitization, allergic disease, eczema, hereditary risk, infant, prevention, randomized controlled trial, vitamin D, wheeze

## Abstract

Lower vitamin D status at birth and during infancy has been associated with increased incidence of eczema and food allergies. The aim of this study was to investigate the effect of early infancy vitamin D supplementation on allergic disease outcomes in infants at “hereditary risk” of allergic disease, but who had sufficient vitamin D levels at birth. Here, we report the early childhood follow-up to 2.5 years of age of “high-risk” infants who participated in a double-blinded, randomized controlled trial. For inclusion in this trial, late gestation (36–40 weeks) maternal 25-hydroxyvitamin D levels needed to be ≥50 nmol/L. Infants were randomized to either oral vitamin D supplementation of 400 IU/day (*n* = 97) or a placebo (*n* = 98) for the first six months of life. Vitamin D levels and allergic disease outcomes were followed up. There were no statistically significant differences in incidence of any medically diagnosed allergic disease outcomes or allergen sensitization rates between the vitamin D-supplemented and placebo groups at either 1 year or at 2.5 years of age. In conclusion, for “allergy high-risk” infants who had sufficient vitamin D status at birth, early infancy oral vitamin D supplementation does not appear to reduce the development of early childhood allergic disease.

## 1. Introduction

Allergic disease prevalence has dramatically increased over recent decades at the same time as environmental and lifestyle changes have occurred, suggesting that these are linked [1,2]. One factor that must be considered is a global change from a more outdoor to an increasing indoor lifestyle. Increased reliance on digital technology and associated time indoors for work and relaxation, and subsequently a lack of sunlight exposure, is a global phenomenon applying to all age groups, including pregnant women and young children [3]. It is known that sunlight exposure plays a crucial role in vitamin D synthesis, with all vitamin D requirements able to be provided by cutaneous synthesis under the influence of ultraviolet (UV) light [4]. Yet despite the potential for adequate endogenous production, vitamin D deficiency is endemic in many parts of the world, even in sun-rich regions, affecting all age groups including the early stages of life [5].

Substantial evidence supports a role for vitamin D in the normal functioning of innate and adaptive immunity in addition to mucosal and epidermal barrier function—factors relevant to the development of immune tolerance and allergic outcomes [6]. Further, notably, the prevalence of allergic disease has been associated with increasing distance from the equator where higher rates of vitamin D deficiency generally occur [7,8,9]. Lower vitamin D status at birth and during early childhood have been associated with increased incidence of allergic disease outcomes [10,11,12,13,14]. However, four randomized controlled trials (RCTs) [15,16,17,18] have investigated the effects of maternal vitamin D supplementation during pregnancy on offspring allergy outcomes and a meta-analysis of these RCTs by Garcia-Larsen et al. [19] found no overall effect on the risk of early childhood recurrent wheeze (4 trials, *n* = 1781 participants, with 358 cases of recurrent wheeze), with a relative risk of 0.76 (95% confidence intervals 0.54; 1.08), nor eczema (3 trials, *n* = 1538 participants, with 357 cases of eczema), with a relative risk of 0.92 (95% confidence intervals 0.77; 1.11). These results suggested that rather than the antenatal period, the early post-natal period in early infancy may be potentially the most critical time to ensure adequate vitamin D status. This is particularly important in breastfed infants as the vitamin D content of breast milk is low and reliant on good maternal vitamin D status during lactation [20]. However, in contrast, one RCT examining maternal vitamin D supplementation found increased food allergy by two years of age in children whose mothers had vitamin D supplementation within the first few months of lactation [21].

In this context, we conducted a double-blinded, randomized controlled trial to investigate early infancy vitamin D supplementation in term infants at “hereditary risk” of allergy development, but who had sufficient vitamin D levels at birth. Here, we report the early childhood allergic disease follow-up outcomes of these children to 2.5 years of age.

## 2. Materials and Methods

### 2.1. Study Population and Intervention

Full details of participant screening, randomization, allocation concealment, intervention and placebo products, and infant outcomes to the end of the intervention period at 6 months of age have been previously published [22]. In brief, inclusion criteria included only infants whose mothers had a sufficient maternal 25-hydroxyvitamin D (25(OH)D) serum concentration of ≥50 nmol/L between 36 and 40 weeks’ gestation. All infant participants were “high-risk” for allergic disease development due to a positive family history of at least one parent or sibling diagnosed with allergic disease (asthma, eczema, allergic rhinitis). Enrolment began on 9 October 2012 and ended on 23 January 2017. A total of 195 infants were randomized into the trial, 97 infants allocated to the intervention vitamin D group and 98 infants to the placebo group.

The intervention group received 400 IU (10 μg) of vitamin D3 per day orally (Ddrops Company, Woodbridge, ON, Canada), while the placebo control group received an identical product of palm kernel and coconut oil without vitamin D3 (Ddrops Company, Woodbridge, ON, Canada). These were given to the infants as 1 drop of liquid (0.03 mL) per day. The randomized controlled trial (RCT) was a parallel design with a 1:1 ratio allocation, and double-blinded with participants and investigators masked to group allocations. Randomization was stratified according to a history of maternal allergic disease and the infant’s sex. The intervention period was from birth until six months of age. For compliance monitoring, caregivers completed a diary card to record each day they gave their infant the study product. Following recommendations for infant vitamin D supplementation [23], caregivers were advised to cease administration of the trial product if the infant was consuming >1000 mL/day of vitamin D-fortified infant formula.

Written informed consent was obtained before trial participation and included consent to participate in both the 1 year and 2.5 years of age clinical allergy follow-up assessments. Human Research Ethics Committee approvals were granted by the Princess Margaret Hospital for Children (approval number 1959/EP) and the University of Western Australia (approval number RA/4/1/5566). The trial was registered with the Australian New Zealand Clinical Trials Registry (ACTRN12612000787886).

### 2.2. Allergic Disease Clinical Outcomes Assessments

At 1 and 2.5 years of age, the participating children were assessed by a pediatric clinical immunologist at the Princess Margaret Hospital in Perth, Australia, who was blinded to the intervention group allocation. A structured history and a standardized clinical examination to diagnose any allergic disease were performed. Skin prick testing was undertaken to common food and environmental allergens, including cow’s milk, hen’s egg, peanut, cashew nut, wheat, cod fish, house dust mite (*Dermatophagoides pteronyssinus*), cat and rye grass pollen on the day of assessment. Sensitization was defined as a positive skin prick test (wheal ≥ 3 mm above negative control) to at least one of the allergens assessed. The children were assessed for eczema, wheeze/asthma and food allergy at 1 year and 2.5 years of age. At the 2.5-year appointment only, the participants were also assessed for a medical diagnosis of allergic rhinoconjunctivitis.

Eczema was defined according to the criteria of Hanifin et al. [24] on medical review or when a typical history was taken of an itchy rash distributed to the facial, extensor or flexural surface of the skin following a chronic or fluctuating course. The use of medically prescribed steroids for eczema treatment was also recorded. IgE-mediated food allergy was defined by a history of an immediate (within 90 min) skin rash (urticaria, erythema), angioedema, gastrointestinal symptoms (abdominal pain, vomiting, diarrhea) and/or irritability associated with or without cardiovascular symptoms (collapse) and/or respiratory symptoms (wheeze, stridor, persistent cough, hoarse voice) following the ingestion of a specific food trigger combined with a matching food allergen sensitization. Wheeze was defined by a history of an audible wheeze responding to inhaled beta2-agonists. Asthma was defined in children over two years of age only, with a history of recurrent wheeze (triggered by upper respiratory tract infections, exercise, cold air or allergen exposure) responding to inhaled beta2-agonists and/or use of a preventer for symptom control. In recognition of the difficulties of asthma diagnosis in young children, at 2.5 years of age, the outcomes of wheeze and/or asthma were combined. Allergic rhinoconjunctivitis was defined as a history of sneezing or blocked/runny nose accompanied by itch-watery/red eyes unrelated to an upper respiratory tract infection. When wheeze/asthma or allergic rhinoconjunctivitis was combined with a sensitization to at least one aeroallergen, it was defined as IgE-associated.

### 2.3. Blood Collection and Measurement of 25-Hydroxyvitamin D (25(OH)D) Levels

Cord blood was collected at birth and peripheral blood samples were collected at 3 months, 6 months, 1 year and 2.5 years of age by venipuncture into lithium heparin tubes (Vacuette, Greiner Bio-One GmbH, Kremsmünster, Austria). The 25(OH)D levels were quantified by a competitive Chemiluminescent Immunoassay, automated on the Abbott Architect i2000 (Abbott Laboratories, Abbott Park, Illinois, operated by PathWest Laboratory Medicine, Western Australia). The Abbott Architect i2000 is accredited by the National Association of Testing Authorities for the measurement of 25(OH)D using a two-step incubation process with human serum calibrators. Internal quality control data indicate the coefficient of variation for 25(OH)D as follows: 11.4% at 22 nmol/L; 5.2% at 48 nmol/L; 4.5% at 68 nmol/L; and 4.0% at 90 nmol/L.

To ensure the infant participants did not develop vitamin D deficiency or toxicity during the trial, blood samples collected at 3 months of age were analyzed for 25(OH)D and the results were sent to a pediatric endocrinologist (AS). Caregivers and the participants’ nominated general practitioner were informed if the 3 month of age level was out of the expected normal range, and the caregivers were asked to cease trial product use and consult their general practitioner for advice. The researchers involved in data collection and analysis remained blinded to these test results throughout the trial.

### 2.4. Statistical Methods

Normality of the continuous variables was determined with the Kolmogorov–Smirnov test of normality. For between-group differences of continuous variables, independent samples t-test or Mann–Whitney U test were used. Mean (standard deviation (SD)) and median (interquartile range (IQR)) were reported for parametric and non-parametric data, respectively. Pearson’s Chi-square test or a Fisher exact test were used for between-intervention/placebo group comparisons and clinical outcomes (categorical variables). To evaluate the effect of the intervention/placebo group on clinical allergy outcomes, logistic regression was applied. All tests were two-sided with a *p* < 0.05 considered statistically significant. Statistical analysis was performed using IBM SPSS Statistics for Windows, version 25 (IBM Corp., Armonk, NY, USA).

## 3. Results

### 3.1. Study Population Characteristics

**Figure 1:**
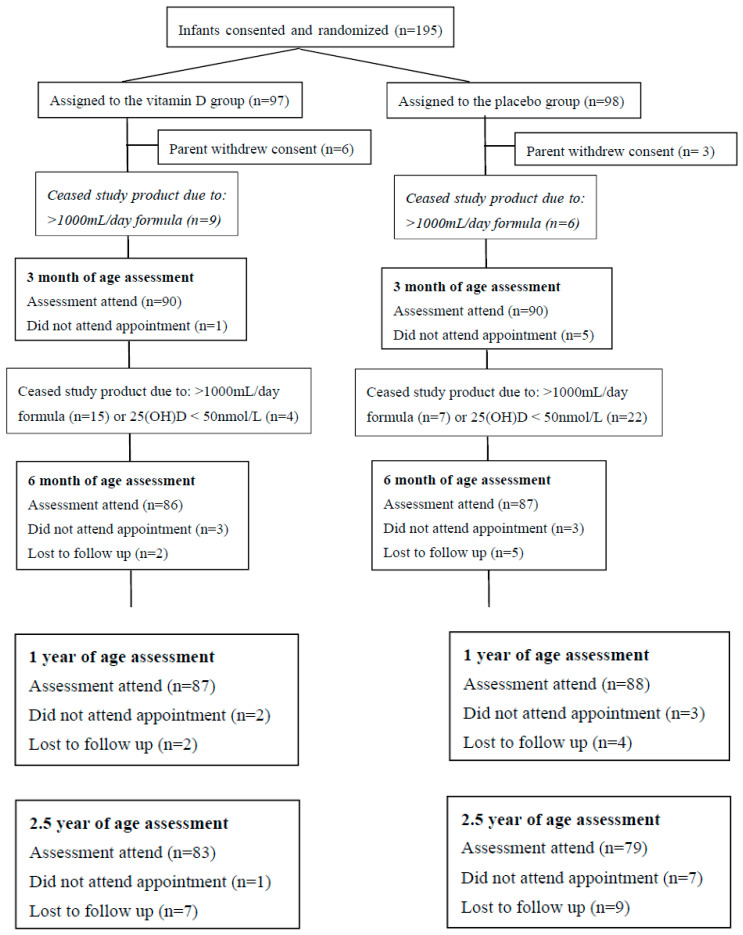
Study flow of participants.

Data collection for the follow-up assessments was completed on 31 January 2019. Participant baseline characteristics have been previously published [22]. Briefly, 53.3% were male infants, 81.5% infants were Caucasian and 9.2% were Asian ethnicity, 61.5% had a vaginal delivery and mean gestational age at birth was 39.2 weeks. During pregnancy, 34.4% of their mothers took vitamin D supplementation. Allergy outcome data were collected on 175/195 (89.7%; 87 from vitamin D group) infants at 1 year of age, and 162/195 (83.1%; 83 from vitamin D group) children at 2.5 years of age, see Figure 1 for full study participant flow details. Nine infants (six in the vitamin D group) were withdrawn by their caregiver(s) during the intervention period, three infants who developed health issues (not allergic disease-related), two infants due to parents becoming too busy and four infants due to parents not wanting to proceed with the blood collections. No infants were withdrawn in the follow-up period from 6 months to 2.5 years of age. One placebo group infant who was thought to be lost to follow-up at 6 months of age could be contacted again and attended their 1 year of age appointment. At 1 year of age, five infants did not attend their appointment due to moving away from the study city, and six infants were lost to follow-up. At 2.5 years of age, eight infants did not attend their appointment (five had moved away and three had both parents now full-time working) and 16 infants were lost to follow-up. No infants experienced any serious adverse events during the intervention or follow-up periods.

### 3.2. Intervention Period Trial Product Compliance and Infant Feeding

During the intervention period from birth until 6 months of age, there were no differences between the two groups regarding compliance in taking the trial product drops. From randomization to 3 months of age, the median (IQR) compliance = 67 (56–75) days in the vitamin D group compared to 68 (59–76) days in the placebo group, *p* = 0.92. From 3 months to 6 months of age, the median (IQR) compliance = 73 (58–84) days in the vitamin D group compared to 75 (50–89) days in the placebo group, *p* = 0.84. From randomization to three months of age, in the vitamin D supplementation intervention group, 83.5% (76/91) of infants were given their trial product drops on ≥5 days per week, compared to 79.2% (76/96) infants in the control group, *p* = 0.57. Between three and six months of age, in the vitamin D supplementation intervention group, 76.9% (70/91) of infants were given their trial product drops on ≥5 days per week, compared to 74.5% (70/94) infants in the control group, *p* = 0.83. As illustrated in Figure 1, there were 9 infants in the vitamin D group and 6 infants in the control group between zero and three months, and 15 infants in the vitamin D group and 7 control group infants between three and six months, who were no longer taking the study product due to consuming >1000 mL/day of vitamin D-fortified infant formula.

After being notified that an infant had an insufficient 25(OH)D level of < 50 nmol/L at 3 months of age, general practitioners recommended either no supplementation or varied dosages of vitamin D supplementation. As illustrated in Figure 1, in the control group, 22 infants ceased the study product use due to a 25(OH)D level of < 50 nmol/L at 3 months of age, but to our knowledge only eight infants commenced supplemental vitamin D use (two infants were supplemented with 200 IU/day for three months, four infants had 400 IU/day for one month, one infant had 400 IU/day for two months and one infant was recommended 1000 IU/day for three months). In the vitamin D-supplemented intervention group, four infants ceased the study product use due to a 25(OH)D level of < 50 nmol/L at 3 months of age, but only one infant was supplemented with 400 IU/day for two months.

Breastfeeding rates were high (98.5% at randomization, 85.2% at 3 months and 70.5% at 6 months of age) and infant formula use was low in our trial participants. There were no differences between the two groups regarding consumption of infant formula. From birth to three months of age, in the vitamin D supplementation intervention group, the median intake of infant formula was 0 mL per day (interquartile range (IQR) 0–250 mL per day), compared to 0 mL per day (IQR 0–235 mL per day) in the control group, *p* = 0.53. Between three to six months of age, in the vitamin D supplementation intervention group, the median intake of infant formula was 3 mL per day (IQR 0–565 mL per day), compared with 17 mL per day (IQR 0–589 mL per day) in the control group, *p* = 0.84. The ingestion of daily infant formula volumes over the preceding three months were also not correlated with vitamin D levels at 3 months of age (r = 0.117, *p* = 0.17) nor at 6 months of age (r = 0.044, *p* = 0.61).

There were no differences between the intervention and placebo groups regarding the age of introduction to solid foods. In the vitamin D group, the median age of commencing solid foods was 5.0 months (IQR 4.3–5.5 months), compared to the control group who had a median age of commencing solid foods of 5.0 months (IQR 4.5–5.5 months), *p* = 0.49.

### 3.3. Longitudinal 25(OH)D Levels over the Course of the Study

As per previously published, baseline cord blood 25(OH)D levels were not significantly different between the two groups [22]. Blood samples were collected from 140 infants (*n* = 68 vitamin D group) at 3 months, 141 infants (*n* = 73 vitamin D group) at 6 months, 154 children (*n* = 79 vitamin D group) at 1 year and 125 children (*n* = 63 vitamin D group) at 2.5 years of age. As previously published, infants in the vitamin D group had significantly higher 25(OH)D levels at 3 and 6 months of age relative to the placebo group [22], however, these differences were not observed to persist beyond the intervention period (Table 1).

### 3.4. Clinical Allergic Disease Outcomes

As expected, the most common medically diagnosed allergic disease in this “high-risk” population was eczema, with an overall incidence of 51/175 (29.1%) infants at 1 year of age and 59/161 (36.6%) children at 2.5 years of age. By 1 year of age, food allergy affected 11/175 (6.3%) infants and wheeze symptoms were diagnosed in 37/175 (21.1%) infants. Positive allergen sensitization to food or environmental allergens was found in 26/167 (15.6%) infants at 1 year and in 31/136 (22.8%) children at 2.5 years of age using skin prick testing. As summarized in Table 2, there were no statistically significant differences in incidence for any of the medically diagnosed allergic disease outcomes or allergen sensitization rates between the vitamin D-supplemented and placebo groups at either 1 year or at 2.5 years of age. The use of prescription steroids for eczema treatment (as a marker for eczema severity) also did not differ between the groups at either 1 year of age with 9.2% (8/87) in the vitamin D group compared to 10.2% (9/88) in the placebo group (*p* = 1.00), or at 2.5 years of age with 12.0% (10/83) in the vitamin D group compared to 12.7% (10/79) in the placebo group (*p* = 1.00).

## 4. Discussion

In this RCT designed to investigate the effects of vitamin D supplementation during early infancy in those infants “at-risk” of allergy development, but with sufficient vitamin D status at birth, we found no statistically significant differences in the incidence of allergic diseases over the first 2.5 years of life between the vitamin D intervention and placebo control groups.

Our finding of no statistically significant effect between vitamin D supplementation and medically diagnosed allergic disease over the first 2.5 years of age is consistent with other intervention trials which have been performed investigating the effect of maternal vitamin D supplementation during pregnancy and offspring allergic disease outcomes [15,16,17,18]. Even more importantly, our findings are also consistent with those in the recent infant RCT by Rosendahl et al. [25], where they also found no statistically significant differences in allergic disease outcomes at 1 year between two groups of infants supplemented daily with either 10 (400 IU) or 30 μg (1200 IU) vitamin D from the age of 2 weeks. While our trial investigated the effect of the Australian recommended dose (400 IU) [26] for vitamin D supplementation in infancy versus placebo on allergy outcomes in early childhood, the study by Rosendahl et al. was based on a secondary analysis comparing two different supplemental doses of vitamin D on allergy outcomes in the first year of life. Rosendahl et al. [25] found no statistically significant differences between the vitamin D supplementation groups for any food allergy (OR 1.35; 95% CI 0.75–2.46), atopic eczema (OR 0.72; 95% CI 0.49–1.01) or wheeze (OR 0.94; 95% CI 0.58–1.50) outcomes at 1 year of age. Furthermore, and of concern, Rosendahl et al. [25] found a higher incidence of cow’s milk allergy in infants administered 30 μg (1200 IU) vitamin D compared with the 10 μg (400 IU) dose (OR 2.23; 95% CI 1.00–4.96), indicating a possible adverse effect of high doses of infant vitamin D supplementation. This has also been identified in the Japanese RCT by Norizoe et al. [21], where maternal vitamin D supplementation (800 IU/day) during early lactation was found to increase the risk of food allergy at 2 years of age.

Due to the exclusion criteria for our RCT of infants with maternal 25(OH)D levels of <50 nmol/L in late pregnancy, it is unclear whether the null findings of this trial would also apply to infants born at higher risk of vitamin D deficiency. However, following international guidelines [27,28], we, along with our Institutional Ethical Review Committee, did not think that it is ethical to allow infants to be knowingly vitamin D-deficient without treatment. Thus, the inclusion of only infants whose mothers were vitamin D-sufficient in late gestation, and the regular three-month testing of vitamin D status within the intervention period of this trial minimized the number and period of time any infant had a deficient vitamin D status. This may have influenced the outcome of our results and explain why we found no differences between the vitamin D intervention group compared to the placebo group. We chose to supplement only the first six months of infancy, but acknowledge that a longer intervention period throughout infancy, and even into early childhood, may have resulted in differences in vitamin D status and in allergy outcomes between the groups at 1 and 2.5 years of age. We also acknowledge as a limitation of this trial, that clinical allergic disease outcomes are a secondary analysis and that our trial sample size was not computed for these clinical allergy outcomes, but rather our sample size was powered to detect potential differences in immune function outcomes at 6 months of age, as per previously published [22].

In our RCT and in that of Rosendahl et al. [25], both involving infants born at term gestation, we did not find any significant benefit of infant vitamin D supplementation on reducing the incidence of medically diagnosed wheeze at 1 year of age. This contrasts with the results at 1 year of age from a recently published paper by Hibbs et al. [29], where preterm black infants (mean gestational age of 33 weeks) were randomized to vitamin D supplementation of 400 IU/day until 6 months of age, and were found to have reduced recurrent wheezing (relative risk 0.66; 95% CI 0.47 to 0.94) compared with a placebo. It needs to be considered that these results were in preterm infants of a different ethnic group and based on parental report by questionnaire rather than medical diagnosis which can lead to misclassification. Hibbs et al. [26] excluded infants who had a 25(OH)D concentration less than 37.5 nmol/L initially, and then lowered this to 25 nmol/L during the trial recruitment period to improve generalizability of the sample and to improve accrual, as it was noted that the prior level of 37.5 nmol/L was excluding a high number of otherwise healthy asymptomatic neonates. In agreement with our RCT results, Hibbs et al. [26] found no significant differences for the most commonly seen allergic diseases in early childhood, eczema nor food allergy between their intervention and control groups. The effect of preterm infant vitamin D supplementation on reduced wheeze outcomes reported by Hibbs et al. [29] appears to relate to the concept previously suggested that vitamin D may have a role in fetal lung development [30], rather than on the development of allergic disease outcomes. This effect may have been found due to the inclusion of some vitamin D-insufficient infants. Furthermore, other antecedents of preterm birth including inflammation, maternal smoking, metabolic derangement, T-cell development and airway reactivity could potentially have had an impact on wheeze outcomes in premature children [31] and were not considered in the study by Hibbs et al. [29].

A limitation of our RCT was that the sample size was powered for immunological outcomes, rather than allergy clinical outcomes. Hence, we eagerly await with interest the clinical allergic disease outcomes from current RCTs investigating infant vitamin D supplementation with the same intervention dose (400 IU/day) in Melbourne, Australia (ANZCTR12614000334606) and in Chiba, Japan (UMIN 000034864). In addition, as these current trials are not only including infants who had sufficient vitamin D status at birth, the potential influence of antenatal vitamin D deficiency may also be able to be examined in these larger current RCTs.

It is interesting that multiple observational studies have previously found that lower vitamin D status at birth, and during infancy, has been associated with increased incidence of early childhood allergic disease [10,11,12,13,14]. This may have been a result of antenatal and/or postnatal vitamin D deficiency, which has not been able to have been investigated in RCTs like our trial reported here, where maternal vitamin D deficiency was an exclusion criterion, and for safety and ethical reasons, any identified infant vitamin D deficiency was treated.

Another potential explanation, related to the associations found in many observational studies between increasing distance from the equator and increasing incidence of allergic diseases is that vitamin D status could be a coincidental marker of ultraviolet (UV) light exposure. UV light may be having a direct effect on the development of allergic disease as well as having a role in vitamin D synthesis. This theory is supported by animal study data showing that UV radiation can cause antigen-specific regulatory T-cell and dendritic cell expansion, causing systemic immunosuppression irrespective of vitamin D status [32,33,34,35]. Moreover, mast cells [36] and regulatory B-cells affecting dendritic cell-mediated T-cell activation are also involved in UV exposure-triggered immunosuppression [37]. A study in mice by Gorman et al. [38] demonstrated that 25(OH)D3 was not essential in mediating the immunosuppressive effects of erythemal UV radiation on contact hypersensitivity responses. Bioactive molecules, such as urocanic acid [39,40] and nitric oxide [41], which are released after sunlight exposure from the skin into the circulation, have been proposed to mediate long-lasting effects on immune function. We suggest that future research closely monitor UV light exposure, both maternal during pregnancy and throughout infancy, to further advance our knowledge regarding our understanding and future recommendations in this field.

## 5. Conclusions

For “high-risk” infants who had sufficient vitamin D status at birth, early infancy oral vitamin D supplementation does not appear to reduce the development of early childhood allergic diseases, however, larger RCTs are required to confirm this. We also do however recommend more research on the possible effects of early life UV light exposure on the development of allergic disease outcomes.

## Figures and Tables

**Table 1:** Longitudinal 25-hydroxyvitamin D (nmol/L) levels over the course of the study. The randomized controlled trial intervention phase was 0–6 months of age. Values are mean (standard deviation) unless otherwise stated. Bold number means significant.

Time Point	Vitamin D Group	Placebo Group	*p*-Value
Cord Blood	67.8 (17.5)	61.1 (14.2)	0.17
3 months of age	83.2 (27.8)	59.2 (22.7)	**<0.01**
6 months of age	93.1 (28.7)	82.0 (27.9)	**0.02**
1 year of age	78.4 (21.9)	81.2 (23.5)	0.45
2.5 years of age ^1^	79.0 (69.0–110.0)	74.0 (60.0–86.5)	0.19

^1^ median (inter-quartile range).

**Table 2:** Comparison between vitamin D-supplemented and placebo groups for medically diagnosed allergic disease outcomes and allergen sensitization rates at 1 and 2.5 years of age. Values are participant numbers (percentages). Abbreviations: CI = confidence interval.

Allergic Disease	Vitamin D Group	Placebo Group	Relative Risk (95% CI)	*p*-Value
Eczema at 1 year	30/87 (34.5)	21/88 (23.9)	1.45 (0.90–2.32)	0.17
Eczema at 2.5 years	33/83 (39.8)	26/78 (33.3)	1.19 (0.79–1.80)	0.50
Food allergy at 1 year	6/87 (6.9)	5/88 (5.7)	1.21 (0.39–3.83)	0.98
Food allergy at 2.5 years	3/83 (3.6)	6/79 (7.6)	0.48 (0.12–1.84)	0.45
Wheeze at 1 year	23/87 (26.4)	14/88 (15.9)	1.66 (0.92–3.01)	0.13
Asthma and/or wheeze at 2.5 years	25/83 (30.1)	18/79 (22.8)	1.32 (0.79–2.23)	0.38
Allergic rhinitis at 2.5 years	16/83(19.3)	11/78 (14.1)	1.37 (0.68–2.76)	0.51
Sensitization at 1 year	10/85 (11.8)	16/82 (19.5)	0.60 (0.29–1.25)	0.24
Sensitization at 2.5 years	17/69 (24.6)	14/67 (20.9)	1.18 (0.63–2.20)	0.75
